# Give or Take: Semantic Priming from Sentences to Two-Digit Operations

**DOI:** 10.3390/brainsci15060662

**Published:** 2025-06-19

**Authors:** Miguel Ayala-Cuesta, Sofía Castro, Pedro Macizo

**Affiliations:** 1 Mind, Brain and Behavior Research Centre (CIMCYC), 18011 Granada, Spain; miguelayala@ugr.es; 2 Department of Experimental Psychology, University of Granada, 18071 Granada, Spain; 3 Institute of Psychology, Jagiellonian University, 31-007 Kraków, Poland; sofia.gonzalez.castro@doctoral.uj.edu.pl

**Keywords:** semantics, language, mathematics

## Abstract

Objectives: The objective of this study was to assess the potential existence of shared semantics between linguistic (e.g., reading a sentence) and numerical information (e.g., performing an arithmetic operation). Methods: To evaluate this proposal, we devised a paradigm with blocks of two trials. In the first trial, participants were presented with sentences containing verbs that conveyed either an increase (e.g., “to give”) or a decrease (e.g., “to take away”). In the subsequent trial, participants were required to perform additions (e.g., 61 + 1) and subtractions (e.g., 52 − 4). We hypothesized that addition and subtraction would exhibit shared semantic processing with sentences denoting increase and decrease, respectively, resulting in cross-domain effects. Results: Participants exhibited enhanced speed and accuracy in addition problem-solving when preceded by increase sentences, whereas subtractions were solved with higher accuracy when preceded by decrease sentences. Moreover, these effects were found to be subject to modulation by the complexity of the numerical operation. Conclusions: The findings of this study support the hypothesis that there is a shared semantic processing between language and mathematics.

## 1. Introduction

Historically, language and mathematics have been considered as discrete domains of investigation (e.g., [1,2] for language; [3,4] for mathematics). However, recent findings in cognitive neuroscience and experimental psychology point to the possibility that these two cognitive domains are at least partially interconnected [5]. Several intuitive factors lend credibility to this suggestion. For example, both language and mathematics require the use of symbols such as letters and numbers, which are fundamental to the creation of words and arithmetic problems, respectively. Furthermore, the foundations of mathematical competence can be rooted in the abstraction of linguistic procedures [6]. Consequently, the discipline of mathematics depends on the existence of relationships between numerical symbols and words [7].

A body of research suggests that the acquisition of certain mathematical concepts may be influenced by the language in which they are learned. For example, Dehaene et al. [8] proposed that exact arithmetic (e.g., 3 × 2 = 6) is typically learned in a single language, making its resolution in an alternative language challenging (see also [5,9]). Furthermore, the discipline of language could be viewed through a mathematical lens, often invoking concepts such as number, quantity, numerical distance, or units of measurement [5]. Moreover, any communicated message requires the correct combination of addition and/or subtraction of letters, words, and phrases to achieve a coherent meaning. Also, common neuroanatomical underpinnings support both language and mathematics [3,8,10,11]. For example, the bilateral intraparietal cortex (IPS) appears to be sensitive to the processing of numerical (e.g., magnitude) and linguistic content [12] (however, see [13,14] for data against a potential overlap between the language network and mathematics). The suggestion of shared functionality between language and mathematics seems quite tenable. The hurdle, however, is to identify the exact processes that are shared between these two cognitive domains. The aim of this study is to provide evidence for the notion of shared processing between language and arithmetic, with particular emphasis on the parallels between sentences denoting increase/decrease and the operations of addition/subtraction.

Researchers intrigued by the potential overlap between language and arithmetic have often used structural priming paradigms [15], a methodological tool designed to investigate whether the response to a particular stimulus is influenced by the structure of a previously presented stimulus. For example, an experiment by Scheepers et al. [16] demonstrated that individuals tend to infer ambiguous sentences that use a syntactic structure that is consistent with a previously solved mathematical equation. In addition, Scheepers and Sturt [17] discovered increased efficiency in solving mathematical equations when their structure matched the syntactic structure of a preceding sentence. Such syntactic priming effects have been confirmed in various languages, including Chinese [18], Russian [19], French [20], and Dutch [21]. While a significant proportion of studies are based on behavioral experiments, the shared structural principles between language and arithmetic have also been supported by alternative methods, such as the eye-tracking paradigm in the visual world [20].

As outlined above, numerous studies provide evidence for shared syntactic processing between language and mathematics, such as the grouping or linking of constituents in sentences and mathematical problems. This shared functionality across domains appears to extend to other cognitive facets, including semantics. However, current data on shared semantic processing are somewhat sparse and yield inconclusive results. For example, Bassok et al. [22] reported that the automatic activation of an addition result is driven by semantic alignment, that is, the semantic coordination between language and arithmetic. In their study, participants were presented with pairs of categorically related prime words (e.g., “tulips–daisies”) and pairs of unrelated words (e.g., “clowns–ovens”) separated by a “+” sign. Following the presentation of these prime words, two digits (e.g., 2, 3) were presented, one above each word. Participants then had to determine whether a target word or digit had been shown previously (e.g., “Did you see tulips?”; “Did you see 3?”). Their results revealed an interference effect due to the automatic activation of the addition result (i.e., the sum effect [22,23]). Essentially, participants found it more difficult to reject as unrepresented a number that matched the result of a previously processed addition (e.g., rejecting 5 after 2 tulips + 3 daisies) than a number that did not match the addition result (e.g., rejecting 7 after 2 tulips + 3 daisies). Notably, this effect only occurred when the word pairs were categorically related (tulips and daisies) and not when they were unrelated (clowns and ovens).

In another investigation of semantic alignment, Guthormsen et al. [24] used event-related brain potentials (ERPs). Participants were tasked with deriving solutions to arithmetic problems in which numerical operands were tagged with object labels that were either semantically aligned (e.g., 3 tulips + 5 roses) or misaligned (e.g., 3 tulips + 5 vases). They also tested the acceptability of semantically aligned (e.g., 12 tulips/4 vases = 3 tulips per vase) or misaligned (e.g., 12 tulips/3 roses = 4 tulips per rose) sentences. The study examined two ERP components, the N400 and the P600 which, very generally speaking, are associated with semantic and syntactic processing, respectively [25]. The results showed greater N400 and P600 amplitudes in response to semantically misaligned compared to aligned problems. These findings support the argument that individuals intertwine mathematical and linguistic information when there is semantic alignment between elements.

Further evidence for shared semantic processing can be found in a recent study by Castro and Macizo [26]. The authors investigated whether the processing of simple affirmative and negative sentences with a subject-verb-complement structure would facilitate the subsequent processing of additions and subtractions. They argued that when processing an affirmative sentence (e.g., “The square is red”), the information provided by the complement is added to the subject, resulting in a semantic affirmation (square + red). Conversely, when processing a negative sentence (e.g., “The square is not red”), the information provided by the complement would limit the information provided by the subject, resulting in a semantic negation (square–red). Based on these assumptions, affirmative sentences would facilitate the processing of additions and negative sentences would facilitate the processing of subtractions. To test this hypothesis, Castro and Macizo presented participants with sentences and arithmetic problems in blocks of two trials. In the first trial, an affirmative sentence (e.g., “The square is red”) or a negative sentence (e.g., “The square is not red”) appeared on the screen, followed by two pictures (e.g., a red square and a green square). Participants were asked to select the picture that corresponded to the meaning of the sentence. Then, an addition or subtraction (e.g., 5 + 3; 5 − 3) was presented, followed by two alternative outcomes. Participants had to choose the correct result. When participants solved difficult subtraction problems, the results showed semantic priming, i.e., subtraction was solved faster after negative than after positive sentences. There was also a tendency for more efficient processing of sums preceded by positive versus negative sentences (although the latter effect did not reach statistical significance). This pattern of results further suggests the existence of shared semantic processing across domains, at least for the processing of semantic negation.

While the evidence presented so far largely supports the existence of shared semantic processing between language and arithmetic, there is evidence that deviates from this direction. A study conducted by Ronasi et al. [27] investigated the effects of bidirectional semantic priming across domains. Their findings indicated a lack of cross-domain semantic priming. The researchers paired additions with exception phrases (EPs) containing negative quantifiers (e.g., “Nobody except John”) and juxtaposed subtractions with EPs containing positive quantifiers (e.g., “Everyone except John”). Participants were asked to judge whether the sentences were meaningful or nonsensical, and then to judge the accuracy of the results of an addition or subtraction problem (e.g., 33 + 8 = 41; 32 − 4 = 28). The prediction was that EPs with positive quantifiers would prime subtraction and those with negative quantifiers would prime addition. However, no semantic priming was observed, suggesting that, at least for EPs with positive/negative quantifiers, there is no shared processing.

The findings reported by Ronasi et al. [27] are not isolated. There are other studies that have also failed to find evidence for shared semantic processing across domains. For example, Monti et al. [28] investigated the effect of language on the cognitive processing of hierarchical algebraic structures using functional magnetic resonance imaging (fMRI). In their study, participants had to determine whether pairs of linguistic arguments (e.g., “Z was paid X by Y”; “It was X that Y paid Z”) and pairs of algebraic arguments (e.g., “X minus Y is greater than Z”; “Z plus Y is less than X”) were synonymous (i.e., they expressed the same concept but were phrased differently) or dissimilar (i.e., they expressed different ideas). The results showed that linguistic arguments activated left perisylvian regions typically associated with language processing, whereas the arithmetic task engaged bilateral parietal brain regions typically associated with numerical magnitude processing. Consequently, these results suggest that the brain regions involved in processing algebraic arguments in language and arithmetic are dissociable. Similar results have been reported in other neuroimaging studies [29] and in studies of patients with language impairment [30]. In these studies, no brain regions associated with language processing were activated during the processing of arithmetic information, further supporting the dissociation between these cognitive domains.

The range of research outlined above highlights the existence of joint syntactic processing in the language and arithmetic domains [16,17,18,19,20,21]. However, the landscape of evidence for shared semantic processing presents a more ambiguous picture. A selection of studies heralds the presence of semantic facilitation across domains [22,24,26,31,32], while others point to a clear dissociation [27,28,29,30]. Our investigation was specifically designed to shed light on these contrasting lines of evidence.

### The Current Study

In the present investigation, conducted in Spanish, our goal was to evaluate the possible semantic overlap between language and mathematics. In contrast to previous studies, in which evidence for or against this overlap was observed, in our study, we explored a new semantic aspect, i.e., the concepts of increase and decrease. According to the Oxford English Dictionary [33], the term “increase” encapsulates “the act, process, or instance of increasing or making larger; expansion, growth, enlargement, extension,” while “decrease” signifies “the process of diminishing; reduction, contraction, decline, tapering off; the resulting state of this process”. Within the framework of language, the essence of numerous verbs embodies the concept of “increase” (e.g., to bestow, to present, to convey, etc.) and “decrease” (e.g., to withdraw, to pilfer, to seize, etc.). For example, the semantic interpretation of the phrase “The boy was given the doll” (*Al niño le dieron la muñeca* in Spanish) implies that the noun phrase (boy) acquires the complement (doll), thereby incorporating the semantic representation of increase. Conversely, the semantic interpretation of the phrase “The girl had the flowers taken away” (*A la niña le quitaron las flores* in Spanish) suggests that the complement (flowers) is being subtracted from the noun phrase (girl), thus embracing the semantic representation of decrease. The identical semantic nuances would be mirrored in the arithmetic domain, where additions correspond to the concept of increase and subtractions correspond to the concept of decrease.

To test these hypotheses, we devised an experimental design consisting of blocks of two-trials. In the first trial, sentences were presented that indicated an increase (e.g., “The boy got the doll”) and a decrease (e.g., “The girl got the flowers taken away”). Two pictures were then presented, and participants were instructed to select the picture that accurately represented the sentence. In the subsequent second trial, arithmetic problems in the form of additions and subtractions were presented, accompanied by two possible answers (e.g., the numbers 94 and 92 following the subtraction problem 97 − 5). Participants were then asked to choose the correct answer.

A secondary goal of our study was to explore the possibility that the difficulty level of arithmetic tasks may influence the manifestation of cross-domain relations between language and arithmetic [26,34]. The performance of complex versus simple arithmetic operations requires a greater degree of cognitive effort, so participants may be inclined to use the semantic cues provided by the preceding sentence to reduce the processing demands of the arithmetic task.

To adjust for the difficulty of the arithmetic problems, we manipulated the numerical distance between the two possible outcomes for the addition and subtraction problems (as in [26]). Extensive research has validated the idea that processing pairs of numbers becomes progressively more difficult as the numerical distance between them decreases [35,36,37,38,39].

In our study, we classified arithmetic problems (e.g., 61 + 5) followed by two alternative outcomes with a small numerical distance (e.g., the correct outcome 66 and the incorrect outcome 64, a numerical distance of 2) as hard problems. Conversely, arithmetic problems followed by two alternative outcomes with a larger numerical distance (e.g., 66 and 62, a numerical distance of 4) were classified as easy problems.

We expected the following results: If the semantic notions of increase and decrease are intertwined from language to arithmetic, we expect to observe facilitation effects characterized by faster response times and improved accuracy. These effects should be present for additions following sentences that use verbs indicating increase as opposed to those indicating decrease, and similarly for subtractions following sentences that use verbs indicating decrease as opposed to those indicating increase.

Furthermore, if the cognitive load imposed by the arithmetic task influences cross-domain semantic modulation, then these facilitation effects should be found when solving more complex arithmetic operations (close distance condition). In the case of far condition problems, participants would respond easily and would not likely require the help of using information from the prior trial. Thus, no priming effects from language to arithmetic are expected during the resolution of easy problems. Pre-registration details for this study are available at https://osf.io/yraq2?view_only=e426bee9eec44841827a94b22a2b5d1a (accessed on 21 July 2020).

## 2. Materials and Methods

### 2.1. Participants

We enlisted fifty participants from the University of Granada, all native Spanish speakers, with an average age of 21.50 years (*SD* = 2.60) and predominantly female (34 out of 50). To determine the sample size for the design used in the current study, we conducted an a priori power analysis using G*Power 3.1.9.7 [40]. For an effect size (*d* Cohen) of 0.25 and a power of 0.95, G*Power recommended a minimum number of 35 participants. Thus, the sample of participants was sufficient to evaluate the initial hypotheses.

Participants consented in writing to partake in the study and were provided with monetary compensation for their involvement. All the procedures implemented during this study adhered to the 1964 Helsinki Declaration and subsequent amendments. The Human Research Committee at the University of Granada granted approval for the study ID 957/CEIH/2019.

### 2.2. Design and Materials

The stimuli, and experimental task used in the current study are freely available at https://osf.io/8mncr/?view_only=6d03334f85b84bb68f9c29e1c1e75ab4 (accessed on 21 May 2023).

The main design of this experiment was a 2 × 2 within-participants factorial design. The independent variables were sentence type (increase or decrease) and operation type (addition or subtraction) (see Table 1).

Beyond the primary experimental design, we also varied the numerical distance (either close or far) between the correct and incorrect proposed outcomes for the arithmetic tasks. This factor was distributed across the problems of the arithmetic tasks. In the near condition, the incorrect result was calculated by adding or subtracting two from the correct result (e.g., for 61 + 5; the correct result = 66, the incorrect result = 64). Conversely, in the far condition, the incorrect result was derived by either adding or subtracting four from the correct result (e.g., for 42 − 8; the correct result = 34, the incorrect result = 38). The experiment was designed using E-Prime 2.0 software [41]. Each block was divided into two trials: an initial sentence trial (Trial 1) followed by an operation trial (Trial 2).

**Trial 1**. To construct the sentences, we chose (a) 10 Spanish verbs (see Table 2 for all Spanish verbs and their English translations), five representative of the increase condition (e.g., “to receive”) and five emblematic of the decrease condition (e.g., “to remove”), (b) 10 direct objects, namely camera, book, lamp, doll, bucket, shoe, watch, telephone, balloon, and flowers, and (c) 2 indirect objects, namely girl and boy. This fusion of 10 verbs, 10 direct objects, and 2 indirect objects culminated in a set of 200 experimental sentences. To ensure that each arithmetic task (addition and subtraction) followed an equal number of sentences, we duplicated the set of sentences, resulting in a total of 400 sentences, 200 to precede additions and 200 to precede subtractions.

To ensure that the verbs in the increase and decrease conditions differed only in their semantic content, the lexical properties of the two types of verbs in Spanish were matched (B-Pal database [42]) (see Table 2). There were no differences in the lexical frequency (per one million count) between increase (*M* = 55.89, *SD* = 108.09) and decrease verbs (*M* = 5.64, *SD* = 5.63), *t*(8) = 1.03, *p* = 0.329. The length in the number of letters was matched in increase (*M* = 7.80, *SD* = 3.27) and decrease verbs (*M* = 7.20, *SD* = 1.64), *t*(8) = 0.36, *p* = 0.706. The orthographic neighborhood was equated in increase (*M* = 2.60, *SD* = 3.20) and decrease verbs (*M* = 1.40, *SD* = 2.609), *t*(8) = 0.65, *p* = 0.534. Finally, the phonological neighborhood was similar in the increase (*M* = 3.00, *SD* = 3.16) and the decrease condition (*M* = 2.60, *SD* = 3.28), *t*(8) = 0.19, *p* = 0.849.

**Figure 1:**
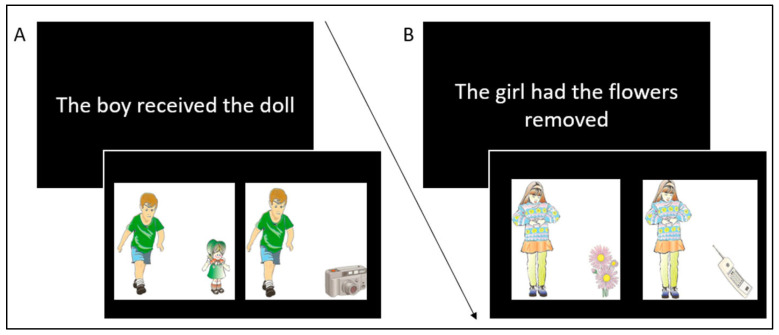
Example trial of the linguistic task (Trial 1). Sentences were presented in Spanish. Panel (**A**): Sentence representing increase. Panel (**B**): Sentence representing decrease. The arrow illustrates the sequence in which the stimuli were presented in Trial 1.

Each sentence was followed by a pair of pictures placed on either side of the screen (see Figure 1).

For increase sentences (e.g., “The boy got the doll”), one image authentically represented the meaning of the sentence (e.g., a boy with a doll, the correct response), while the other image included an unrelated object not mentioned in the sentence (e.g., a boy with a camera, the incorrect response). Conversely, for decrease sentences (e.g., “The girl had the flowers removed”), one image depicted the girl with the object that was supposedly removed according to the sentence (e.g., a girl with flowers, the incorrect response because the notion of “removed” is not depicted), while another image depicted the girl with another object (e.g., a girl with a telephone, the correct response because it does not match the semantic information in the sentence). The location of the correct picture (alternately on the right and left side of the screen in an equal number of trials) was randomized across the experiment. The illustrations were generated by merging an image of a boy or a girl with one of the 10 objects mentioned above, resulting in 20 unique images. Incorrect pictures were pseudo-randomly selected from the pool of pictures that contradicted the meaning of the sentence. All images were adapted from the Illinois Test of Psycholinguistic Abilities (ITPA [43]). The full set of stimuli for Trial 1 can be found in Appendix A (Table A1 and Table A2).

It should be noted that the visual stimuli associated with the sentences in the decrease condition (e.g., “The girl had the flowers removed”) could have been generated by presenting the subject with an object not specified in the sentence (e.g., the girl with a telephone, an incorrect response) or by presenting only the subject (e.g., the girl) in isolation, without any additional object (a correct response). However, this particular form of stimulus presentation has several limitations: (a) it may introduce a bias, as participants may be inclined to focus on the picture depicting a subject without an object immediately after reading a negative sentence in the decrease condition. That is, they might skip processing the alternative picture altogether. (b) The visual complexity of the correct image (i.e., the girl without an object) and the incorrect image (e.g., the girl with a phone) would not be comparable, with the correct image being less complex and easier to process than the incorrect one. (c) If we were to consider a subject without an object as a correct response in the decrease condition sentences, this response alternative would be different from the other response alternatives in both the increase and decrease sentences, which would make it more salient. For these reasons, we decided to use the paradigm as described above.

**Trial 2**. The arithmetic operations were derived from an existing database containing 480 operations [44]. Addition and subtraction problems were assembled by randomly pairing two-digit numbers (ranging from 11 to 99) as the first operand with one-digit numbers (ranging from 1 to 9) as the second operand. Operations that resulted in a number containing the digit zero (e.g., 37 + 3 = 40) or negative numbers were excluded. For the purpose of this study, 400 operations were randomly selected from the database (200 additions and 200 subtractions). Half of these additions and subtractions were assigned to the ‘close’ condition (where the numerical distance between the two suggested results is 2). The remaining operations were assigned to the ‘far’ condition (where the numerical distance between the two suggested results is 4). The position of the correct result, which was equally divided between the right and left side of the screen, was randomized across trials. The full set of operations used in this experiment can be found in the Appendix A (Table A3).

The combination of sentences in Trial 1 and operations in Trial 2 resulted in a total of four different combinations: (a) 100 additions following sentences indicating increase, (b) 100 additions following sentences indicating decrease, (c) 100 subtractions following sentences indicating increase, and (d) 100 subtractions following sentences indicating decrease. In each of these four scenarios, half of the operations (50) were assigned to the ‘close’ numerical distance condition, while the remaining half (50) were assigned to the ‘far’ numerical distance condition.

### 2.3. Procedure

Before the experiment began, participants were familiarized with the images and the experimental task. All object images were displayed, each labeled with its corresponding name. Participants advanced to the next image by pressing the space bar. Five practice blocks of two trials each were then introduced, preceding 400 experimental blocks of two trials. Each block began with the first trial in which a fixation dot (*****) was displayed for 500 ms, followed by a sentence for 1000 ms. After the sentence disappeared, two images were presented side by side, both matching the gender of the character described in the sentence (i.e., boy or girl), but depicting different objects. Participants had to select the image congruent with the sentence by pressing the Z key (if the correct image was on the left side of the screen) or the M key (if it was on the right side of the screen).

After the participant responded, the second trial started with a fixation point displayed for 500 ms, followed by an addition or subtraction problem for 1000 ms. After the problem disappeared, two possible solutions were presented side by side. Participants had to select the correct solution by pressing the Z key (if the correct solution was on the left) or the M key (if it was on the right). The term MODO was then displayed in the center of the screen. At this point, participants were asked to report the strategy they used to solve the operation (i.e., retrieve, count, transform, or “other”) by pressing one of four designated keys. The “mode” question was implemented to ensure that the types of strategies reported by participants to verify the operations in our study were comparable to others reported in previous works [45].

**Figure 2:**
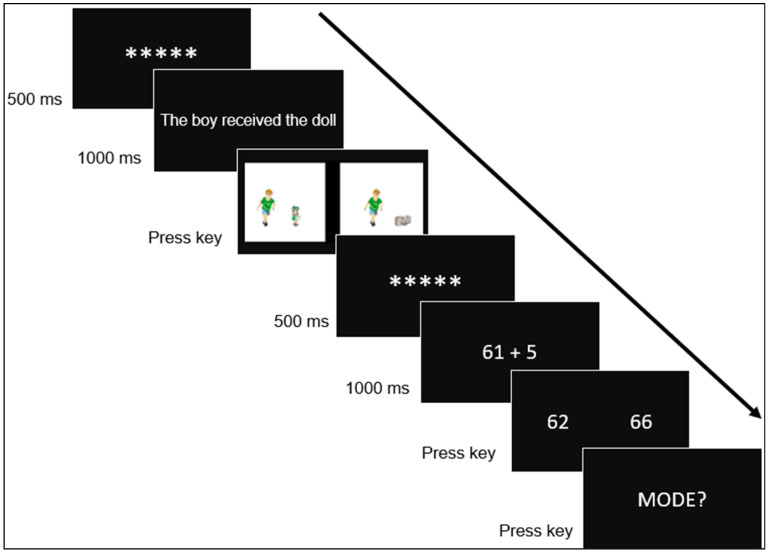
Example of an experimental trial. The arrow illustrates the sequence in which participants perform the task. In Trial 1, a sentence is displayed for 1000 ms, followed by two images. Participants are required to select the image that matches the meaning of the sentence. In Trial 2, an addition or a subtraction is displayed for 1000 ms, followed by two numbers. Participants are required to choose the correct answer for the operation. Finally, participants are required to press a key to indicate how they solved the operation (retrieval, counting procedure, transformation, or other).

Prior to the experiment, participants received a written explanation of the four strategies: Retrieval strategies involve recalling from memory (e.g., “When a problem such as 2 + 4 = is presented, you recall from memory that 6 is the correct answer”). Non-retrieval strategies include counting (e.g., “When presented with a problem such as 2 + 4 =, you mentally count from 2… 3, 4, 5, and 6 to arrive at the answer”), transformation (e.g., “When presented with a problem such as 2 + 4 =, you decompose it into simpler problems, e.g., 2 + 2 + 2”), and other strategies that differ from those previously outlined. These instructions were given to the participants before they started the experimental task to make them aware of the differences between the strategies commonly used to solve arithmetic problems. Subsequently, during the performance of the experimental task, in the second trial of each block (addition and subtraction trials), self-reports of each participant on the strategy used to solve each arithmetic problem were collected. The full experimental procedure is illustrated in Figure 2.

### 2.4. Data Cleaning and Code Availability

Prior to analysis, trials with errors in Trial 1 were removed. Univariate outliers in reaction times (RTs) for Trial 1 were then excluded, following the approach recommended by Tabachnick and Fidell [46]: Raw scores were converted to standard scores (z-scores), and data that fell 3 standard deviations (*SD*) outside of the normal distribution were classified as outliers. After outlier removal, z-scores were recalculated. This filtering process was repeated for ten iterative cycles. For Trial 1, the proportion of outliers was 5.66%.

A similar procedure was used in Trial 2 for the removal of univariate outliers in RTs (9.74%). In total, we retained 75.25% of the original data (15,051 trials) for the priming analysis.

Given the nature of the experiment (priming), the removal of one of the trials (verbal or mathematical task) required the removal of the entire block. In other words, if a sentence was removed, the corresponding operation was also discarded, and vice versa.

All data analyses were performed using the statistics package within the R software environment, version 4.1.1 [47].

The data and analyses conducted in this study are freely available at https://osf.io/8mncr/?view_only=6d03334f85b84bb68f9c29e1c1e75ab4 (accessed on 21 May 2023).

## 3. Results

We first examined the percentage of strategies used to solve the arithmetic problems. This was done in order to confirm that the task (linguistic/arithmetic) used in our study did not change the pattern of strategies usually observed when people solve addition and subtraction problems in an arithmetic context alone. The self-report of strategies indicated that additions were solved primarily by retrieval strategies (62.83%), followed by counting (23.72%), transformation (9.58%), and finally, “other” strategies (3.87%). A comparable proportion of strategies was reported in the context of solving subtractions. Participants indicated that they were solved primarily by retrieval strategies (64.43%), followed by counting (21.19%), transformation (9.88%), and “other” strategies (4.50%). Therefore, the preferred strategy in our study (retrieval, 63%) corroborates the findings of previous research and falls within the range of the retrieval strategy reported by other authors (71% for one-digit operands, as observed by LeFevre et al. [48], and 57% for two-digit sums, as reported by Lemaire and Arnaud [49]. Therefore, the strategic pattern exhibited by the participants in resolving arithmetic problems, which primarily favors retrieval, is consistent with the findings of other investigations. This consistency provides reassurance that the experimental design, comprising blocks of two trials, did not exert an artificial influence on the approach taken by participants to the arithmetic task in this study.

### 3.1. Latency Analysis

A one-way analysis of variance (ANOVA) was performed for the linguistic task (Trial 1), including the type of sentence (increase/decrease) as the independent variable. The results showed that participants were slower when responding to sentences indicating a decrease (*M* = 885 ms, *SE* = 29.4) compared to sentences indicating an increase of information (*M* = 809 ms, *SE* = 29.4), *F*(1, 49) = 74.92, *p* < 0.001, *η*_p_^2^ = 0.78.

A 2 (Numerical distance: close/far) x 2 (Type of sentence: increase/decrease) x 2 (Type of operation: subtraction/addition) ANOVA was performed on Trial 2 (See Appendix B, Table A4 for the results) on the numerical operations. The main effect of numerical distance was significant, *F*(1, 49) = 13.5, *p* < 0.001, *η_p_*^2^ = 0.21, meaning that participants were faster selecting the correct response when the numerical distance between the two results was far (*M* = 860 ms, *SE* = 55.1) compared to when it was close (*M* = 894 ms, *SE* = 55.1). The effect of type of operation was also significant, *F*(1, 49) = 14.47, *p* < 0.001, *η_p_*^2^ = 0.22, meaning that participants were faster at selecting the correct result for additions (*M* = 853 ms, *SE* = 55.3) than subtractions (*M* = 901 ms, *SE* = 55.3).

The Numerical Distance x Type of Operation interaction was significant, *F*(1, 49) = 20.1, *p* < 0.001, *η_p_*^2^ = 0.29. Also, the three-order interaction Numerical Distance x Type of Sentences x Type of Operation was significant, *F*(1, 49) = 12.28, *p* < 0.001, *η_p_*^2^ = 0.20. To explore this interaction, we evaluated the effect of type of sentence and type of operation on each of the numerical distance levels.

When the numerical distance was close (i.e., numerical distance between the two response options for the operation was 2, e.g., numbers 62 and 64 as response alternatives), there was a significant effect of type of operation, so that additions were solved faster (*M* = 853 ms, *SE* = 55.6) than subtractions (*M* = 935 ms, *SE* = 55.6), *F*(1, 49) = 33.42, *p* < 0.001, *η_p_*^2^ = 0.40. Furthermore, we observed a significant interaction between type of sentence and type of operation, *F*(1, 49) = 13.69, *p* < 0.001, *η_p_*^2^ = 0.21. We conducted pairwise comparisons to evaluate if the response times for the additions and subtractions depended on the type of sentence (increase or decrease). To control for multiple comparisons, Bonferroni corrections were applied (adjusted *p* = 0.025). This analysis revealed that participants solved additions faster when they were preceded by increase (*M* = 825 ms, *SE* = 56.2) compared to decrease sentences (*M* = 881 ms, *SE* = 56.2), *t*(188) = 3.43, *p* < 0.001, *d_z_* = 0.25. However, there were no significant differences for subtractions, *t*(188) = −1.08, *p* = 0.280, *d_z_* = 0.07.

**Figure 3:**
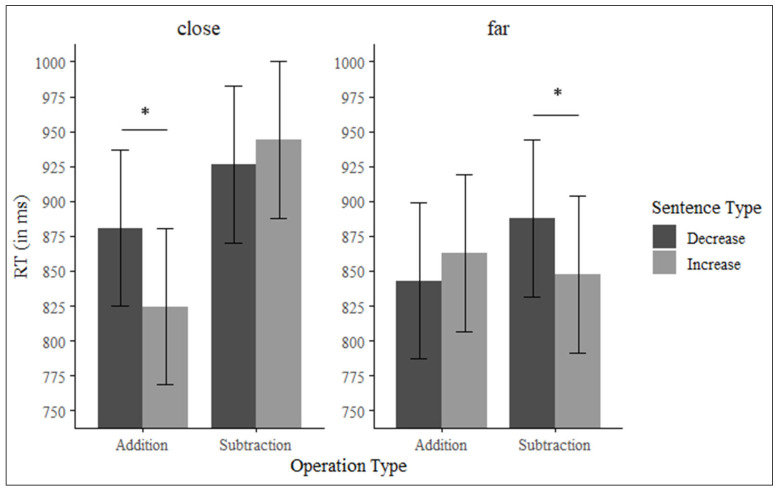
Reaction times for additions and subtractions based on the sentence type and numerical distance. Error bars represent standard error. * *p* < 0.05.

When the numerical distance was far (i.e., numerical distance between the two response options for the operation was 4, e.g., numbers 62 and 66 as response alternatives), the main effect of type of operation was not significant, *F*(1, 49) = 0.91, *p* = 0.346, *η_p_*^2^ = 0.01. However, we found a significant interaction between type of sentence and type of operation, *F*(1, 49) = 4.47, *p* = 0.039, *η_p_*^2^ = 0.08. Bonferroni corrections were also applied to the following pairwise comparisons. Pairwise comparisons revealed that participants solved subtractions faster when preceded by increase (*M* = 848 ms, *SE* = 56.2) versus decrease sentences (*M* = 888 ms, *SE* = 56.2), *t*(188) = 2.44, *p* = 0.015, *d_z_* = 0.17, but there was no significant effect for additions, *t*(188) = −1.22, *p* = 0.222, *d_z_* = 0.08 (see Figure 3).

### 3.2. Accuracy Analysis

The percentage of correct responses in Trial 1 was 94.64% and in Trial 2 was 95.32%. A one-way ANOVA analysis was performed for Trial 1, including the type of sentence (increase/decrease) as independent variable. The results showed higher accuracy for increase (*M* = 96%, *SE* = 0.67) than for decrease sentences (*M* = 93.3%, *SE* = 0.67), *F*(1, 49) = 14.6, *p* < 0.001, *η_p_*^2^ = 0.22.

As in the RT analyses, for Trial 2, we conducted a 2 (Numerical distance: close/far) × 2 (Type of sentence: increase/decrease) × 2 (Type of operation; subtraction/addition) ANOVA (see Appendix B, Table A5 for the results). The main effect of numerical distance was significant, *F*(1, 49) = 44.5, *p* < 0.001, *η_p_*^2^ = 0.47, as well as the main effect of type of operation, *F*(1, 49) = 30.64, *p* < 0.001, *η_p_*^2^ = 0.38. The accuracy was higher for far (*M* = 96.7%, *SE* = 0.65) than close distance operations (*M* = 93.6%, *SE* = 0.65), and for additions (*M* = 96.5%, *SE* = 0.66) than subtractions (*M* = 93.7%, *SE* = 0.66). We also found significant interactions between type of sentence and type of operation, *F*(1, 49) = 5.17, *p* = 0.027, *η_p_*^2^ = 0.09, and between type of operation and numerical distance, *F*(1, 49) = 12.65, *p* < 0.001, *η_p_*^2^ = 0.20. The three-order interaction between numerical distance, type of sentences, and type of operation was also significant, *F*(1, 49) = 5.74, *p* = 0.020, *η_p_*^2^ = 0.10. As in the case of the response times, we analyzed whether the effect Type of Sentence x Type of Operation depended on the numerical distance.

**Figure 4:**
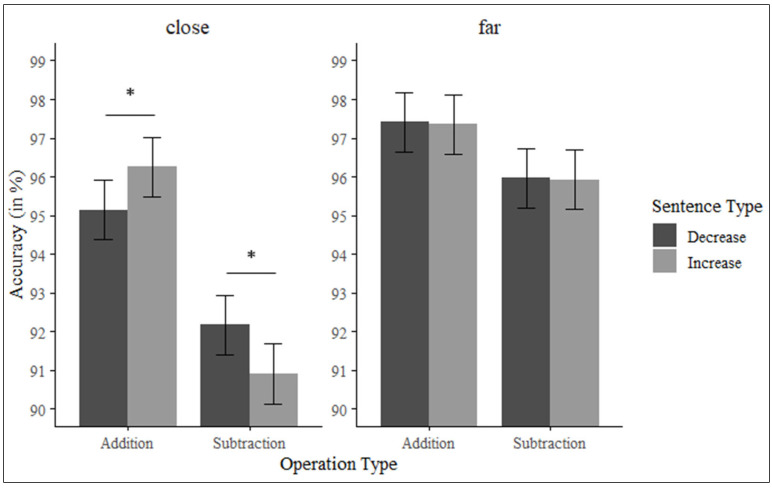
Accuracy for additions and subtractions based on the operation type and numerical distance. Error bars represent standard error. * *p* < 0.05.

When the numerical distance was close, participants were more accurate at solving additions (*M* = 95.7%, *SE* = 0.72) than subtractions (*M* = 91.5%, *SE* = 0.72), *F*(1, 49) = 27.03, *p* < 0.001, *η_p_*^2^ = 0.35. In addition, the interaction between type of sentence and type of operation was significant, *F*(1, 49) = 8.22, *p* = 0.006, *η_p_*^2^ = 0.14. Pairwise comparisons (with Bonferroni correction applied) revealed that additions were solved more accurately when preceded by increase (*M* = 96.3%, *SE* = 0.77) than by decrease sentences (*M* = 95.1%, *SE* = 0.77), *t*(194) = −2.14, *p* = 0.033, *d_z_* = 0.15. Moreover, the accuracy was higher for subtractions preceded by decrease (*M* = 92.2%, *SE* = 0.77) than by increase sentences (*M* = 90.9%, *SE* = 0.77), *t*(194) = −2.43, *p* = 0.015, *d_z_* = 0.17 (See Figure 4).

Lastly, when the numerical distance was far, participants were more accurate with additions (*M* = 97.4%, *SE* = 0.72) than subtractions (*M* = 95.9%, *SE* = 0.72), *F*(1, 49) = 12.66, *p* < 0.001, *η_p_*^2^ = 0.20. However, the interaction between type of sentence and type of operation was not significant, *F* < 1.

The means of the reaction times and the percentage of accuracy obtained in all conditions are shown in Appendix B, Table A6.

In conclusion, findings from this investigation reveal that participants managed addition problems more swiftly and accurately than subtraction ones. However, the operational processing fluctuated based on the nature of the sentence and numerical distance. As hypothesized, during instances of close numerical distance, additions were resolved more rapidly and accurately when followed by sentences signifying increase versus those indicating decrease. Simultaneously, subtraction problems were answered with greater accuracy post-decrease sentences as compared to increase sentences, although reaction times exhibited no notable discrepancies. Yet, in scenarios where numerical distance was far, subtraction tasks were completed more rapidly following increase sentences, contrary to our initial hypothesis.

## 4. Discussion

The examination of common cognitive processing principles spanning diverse disciplines, including language and mathematics, has emerged as a significant area of enquiry in contemporary research. A substantial body of evidence substantiates the occurrence of mutual syntactic processing [16,17,18,19,20,21]. However, findings pertaining to joint semantic processing depict a more intricate landscape. Some studies demonstrate the cross-domain processing of semantic information [22,24,26,31,32], whereas others provide no substantial evidence of such an association [27,28,30]. Consequently, the existing body of evidence concerning the mutual semantic processing between mathematics and language remains inconclusive and somewhat dispersed.

The objective of this study was to elucidate the possible semantic relationship between linguistic and arithmetic processing. To this end, we conducted a thorough examination of whether the semantic notions of ‘increase’ and ‘decrease’ exhibit a parallel role in the processing of language and mathematical tasks. An experimental paradigm was devised whereby sentences conveying either an increase or decrease in meaning acted as priming factor (Trial 1) when followed by addition or subtraction problems (Trial 2). The aim was to ascertain whether the priming sentences influenced the outcome of the subsequent mathematical tasks. Based on a pre-registered premise (https://osf.io/yraq2?view_only=e426bee9eec44841827a94b22a2b5d1a accessed on 21 July 2020), we hypothesized that if the ‘increase’ and ‘decrease’ concepts are coherently utilized across language and mathematics, we would thus expect to observe a facilitative effect, manifesting as swifter response times and improved accuracy for additions succeeding ‘increase’ statements as opposed to ‘decrease’, and similarly for subtractions following ‘decrease’ phrases compared to ‘increase’. To gain further insight into the impact of cognitive burden on cross-domain semantic processing, we introduced variations in task complexity. Building upon the findings of previous research [16,17,26], we hypothesized that any observable priming effects would be amplified during more demanding tasks (i.e., operations involving close numerical distances) relative to simpler tasks (i.e., operations with far numerical distances).

In the first trial, participants were instructed to select an image that was consistent with the sentence they had just read. When presented with a sentence indicating a decrease, participants were required to discard the image featuring the object referenced in the statement and select instead the image displaying the unmentioned object. It is therefore proposed that this selection procedure entails a semantic negation, implying that the processing of decrease sentences could be construed as akin to negative sentences [50], while increase sentences would align with affirmative statements. The results of the linguistic task (Trial 1) demonstrated that sentences indicating an increase were processed more efficiently than those suggesting a decrease, as evidenced by reduced reaction times and enhanced accuracy. These findings are consistent with previous research indicating that negative utterances are associated with increased processing difficulty compared to affirmative utterances [51,52,53,54,55,56].

Although we have argued that the differences between increase and decrease sentences are semantic, syntactic differences cannot be ruled out (i.e., greater syntactic complexity in decrease sentences than in increase sentences). However, the experimental paradigm used in our study does not allow us to confirm a syntactic effect, since any observed effect would coincide with the semantic differences between the two types of sentences.

It is of particular importance to note that the following key findings have been identified in the course of this investigation: The results of the mathematical operations (Trial 2) provided evidence in support of the presence of semantic priming. However, these priming effects were found to be subject to modulation by task complexity. To be more precise, when the numerical distance between the proposed results was close (i.e., 2), participants resolved addition problems more rapidly and accurately when these were introduced by sentences indicating an increase as opposed to a decrease (although the effect size was small, *d_z_* = 0.25). In contrast, the participants solved subtraction problems more accurately when the sentences indicated a decrease rather than an increase. With regard to reaction times in close decrease sentences followed by subtractions, although the interaction between sentence type and subtractions was not significant, as can be seen in Figure 3 (left panel), there was a trend in line with what was expected, suggesting faster responses to subtractions preceded by decrease vs. increase sentences.

One could argue that the numerical distance effect observed in this study is not related to the semantic priming found with close distances, since the numerical distance involves the two response alternatives once participants compute the arithmetic operation. However, we believe that both effects, semantic priming and numerical distance, are related (in fact, an interaction between the two effects was observed). For example, in the close condition, participants would solve sums more quickly if the sentences denoting increase were present prior to the sums. This would ultimately facilitate the subsequent selection of the correct result between the two response alternatives presented.

The results found with problems in the close condition support the hypothesis of shared semantic processing between language and mathematics [22,24]. Furthermore, they align with the evidence suggesting that the resolution of arithmetic operations is modulated by task difficulty [16,17,26]. In instances where the operations were of a complex nature (i.e., with a close numerical distance), the participants demonstrated a clear benefit from the semantic information provided by the preceding sentence (i.e., semantic priming from the sentences to the operations).

Conversely, when the operations were more straightforward to process (i.e., when the numerical distance between result options was larger), no priming was observed between language and arithmetic. This pattern of results is not aligned with our initial hypotheses, but it corroborates the notion of a shared working memory system for language and mathematical processing. In a similar vein, Van de Cavey and Hartsuiker [21] put forth the proposition that language and arithmetic draw upon shared working memory resources, thereby facilitating the integration of information from both domains. In accordance with the concept of shared working memory resources, research has indicated that task difficulty is a contributing factor in the priming effects observed between language and mathematics [57]. Specifically, priming effects are more prominent when a task is more challenging than when it is easier [34,58]. Consequently, when the cognitive load imposed by an easy task on working memory is reduced, individuals are able to successfully perform the task without the need for additional priming facilitation. Conversely, the cognitive demands of a difficult task can result in an overload of working memory, thereby making the benefits of priming more apparent in difficult tasks. This argument provides an explanation for the presence of facilitation effects observed in operations such as addition and subtraction that are challenging to solve, particularly when the proposed result is close to the correct answer. In contrast, for arithmetic operations that are easy to solve, with a significant discrepancy between the proposed result and the correct answer, participants may not require the assistance of a linguistic prime to perform the task efficiently.

However, one limitation of our study was the pattern of results when the numerical distance was far. Specifically, reaction times were slower when participants solved subtraction problems in the decrease condition than in the increase condition. This finding cannot be explained by the idea of shared working memory resources between language and mathematics [21]. According to this theoretical perspective, priming effects between sentences (increase and decrease) and arithmetic operations (additions and subtractions) would not be expected, since arithmetic problems with a large numerical distance would require few processing resources in working memory and could be solved without attending to the linguistic information presented in the first trial. We have no theoretical explanation for this unexpected effect. In any case, it is important to note that this effect was observed only in latencies and not in response accuracy. Thus, when participants solved the subtractions in the far condition, the times were slower for the decrease versus increase sentences, but the response accuracy was equal in both conditions. Future research (including our own) should determine whether this pattern of unexpected results obtained in response latencies but not accuracy analyses is a spurious effect or confirmed.

The findings of our investigation support the hypothesis of a shared semantic system between language and arithmetic [22,24,26]. To be more precise, our findings illustrate the phenomenon of transfer, or semantic priming, from language to arithmetic. Further research should be conducted to ascertain whether these semantic concepts are applicable in reverse across these domains (i.e., from mathematics to language). To this end, mathematical operations would need to serve as primes and sentences as targets. The procedure outlined in this study could be adapted for this purpose, after eliminating the section where participants report their problem-solving strategies (i.e., MODE). This modification would allow for the analysis of reaction times and accuracy in relation to increase and decrease sentences, based on previously solved operations.

Previous studies have provided evidence for [22] and against [27,28,30] shared semantic processing between language and mathematics. The novelty of this study lies in demonstrating that it is possible to find relationships between these two cognitive domains when specifically considering which semantic aspects are shared (the concept of increase and decrease in our study) and using an experimental paradigm that allows capturing these effects (blocks of a linguistic and a numerical trial in our case).

### Implications of the Current Study for Future Lines of Research

The first extension of our line of research would relate to deductive reasoning. More specifically, it would focus on transitive inference (TI), which allows us to derive relationships between elements that have not been previously compared [59,60,61]. TI is closely related to the concept of magnitude and the notions of increase, decrease, addition, and subtraction. For instance, if A is greater than B (A > B) and B is greater than C (B > C), then we can infer that A is greater than C (A > C). This TI is closely linked to the concept of magnitude increase and the solution of addition problems. Conversely, if A is smaller than B (A < B) and B is smaller than C (B < C), then it can be inferred that A is smaller than C (A < C). This type of TI is related to the concept of information decrease and solving subtraction problems. Taking this into account, one could evaluate the following in future experiments: (a) the relationship between linguistic information (first trial) and subsequent TI resolution (second trial); (b) the relationship between arithmetic problem-solving (additions and subtractions in the first trial) and subsequent TI resolution (in the second trial). If linguistic material, arithmetic content, and TI share common semantic information (i.e., magnitude), priming effects between language-TI and arithmetic-TI would be expected. Moreover, the observation in the present study that congruent semantic priming improves arithmetic performance under high-difficulty conditions reflects the same dependency on relational consistency and cognitive load. These parallels suggest that the mechanisms underlying semantic priming and TI reasoning are highly similar.

Second, in our research, we evaluated the differential effect of linguistic information (“increase” and “decrease” sentences) on the subsequent resolution of arithmetic operations (additions and subtractions). Behavioral results showed semantic facilitation effects between sentences and arithmetic operations in the close condition, which occurred when there was semantic alignment between the linguistic and mathematical content (e.g., increase-addition problems and decrease-subtraction problems). Brain-based studies demonstrate a dissociation in the performance of addition and subtraction. For example, Kutter et al. [62] explored the brain mechanisms involved in simple arithmetic operations by recording single-neuron activity from the medial temporal lobe of human participants performing additions and subtractions. The authors found abstract and notation-independent codes for addition and subtraction in neuronal populations, for a review see [63]. Thus, future research could investigate whether the dissociation in neural pathways between additions and subtractions extends to linguistic material conveying increase and decrease information, respectively.

Third, our study’s results can be contextualized within the framework of Bayesian inference and its connection to fuzzy logic with respect to the semantic priming observed in our research. Bayesian inference is a probabilistic method that uses Bayes’ theorem to adjust prior beliefs or probabilities based on new evidence. Fuzzy logic, on the other hand, is designed to handle imprecise or vague information by allowing degrees of membership to sets rather than strict binary membership (either in or out) [64,65]. From a fuzzy logic perspective, the concepts of “increase” and “decrease” employed in our study should not be considered “all or nothing”. Rather, a fuzzy set for “increase” (or “decrease”) could have membership values between 0 and 1. In this system, 0 would represent a lack of “increase” (or “decrease”), while 1 would represent the greatest degree of “increase” (or “decrease”), with values in between representing varying degrees. Thus, according to fuzzy logic and Bayesian inference, the semantic concepts used in our study (increase and decrease of information) should not be considered unitary entities, but rather membership functions over graded semantic spaces.

## 5. Conclusions

In this study, we investigated whether language processing (e.g., sentence comprehension) and mathematical processing (e.g., solving arithmetic problems) share a common semantic representation of the concepts of increase and decrease. The answer is yes, but with restrictions. The results revealed that solving addition and subtraction problems was facilitated when there was semantic alignment between language and mathematics (e.g., increase sentences and addition, decrease sentences and subtraction). However, this effect depended on the cognitive load of the arithmetic task. Specifically, semantic facilitation was evident under high-load conditions. Thus, we can conclude that shared semantics exists between language and mathematics, which becomes more evident as the task difficulty increases (i.e., high cognitive load).

## Figures and Tables

**Table 1:** Experimental conditions.

Sentence Type	Operation Type	
	Addition	Subtraction
Increase sentence	Increase + Addition (congruent)	Increase + Subtraction (incongruent)
Decrease sentence	Decrease + Addition (incongruent)	Decrease + Subtraction (congruent)

**Table 2:** Lexical properties of the verbs used in the study.

Verbs	Lexical Frequency	Length	Orthographic Neighbors	Phonological Neighbors
Increase				
dar (to give)	249.11	3	8	8
regalar (to gift)	4.46	7	3	4
entregar (to deliver)	12.5	8	1	2
proporcionar (to provide)	10.71	12	1	1
adjudicar (to assign)	2.68	9	0	0
Decrease				
quitar (take away)	13.75	6	1	3
robar (to steal)	9.46	5	6	8
arrebatar (to snatch)	3.21	9	0	0
sustraer (to rob)	1.43	8	0	0
requisar (to requisition)	0.54	8	0	2

The verbs were presented in Spanish, with the English translation provided in brackets. The lexical properties were computed in Spanish. Lexical frequency is obtained per one million count, with length considered in number of letters. Orthographic neighbors are determined by counting the number of words that can be formed by substituting a single letter at any of the letter positions within the string. Phonological neighbors are similar to those for the orthographic neighborhood but including deletion and addition neighbors [42].

## Data Availability

Publicly available datasets were analyzed in this study. This data can be found here: https://osf.io/8mncr/?view_only=6d03334f85b84bb68f9c29e1c1e75ab4 (accessed on 21 May 2023).

## Appendix A. Stimulus Used in the Experiment

**Table A1:** Lists of verbs used in the sentences.

Verbs Denoting Increase	Verbs Denoting Decrease
dieron (gave)	quitaron (took away)
regalaron (gifted)	robaron (stole)
entregaron (delivered)	arrebataron (snatched)
proporcionaron (provided)	sustrajeron (robbed)
adjudicaron (assigned)	requisaron (requisitioned)

**Table A2:** List of objects used in the language task.

Name	Image	Name	Image
Niño (Boy)		Niña (Girl)	
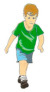
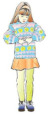
Cámara (Camera)		Libro (Book)	
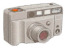
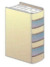
Cubo (Bucket)		Muñeca (Doll)	
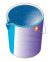
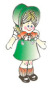
Flores (Flowers)		Reloj (Watch)	
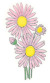
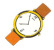
Globo (Balloon)		Teléfono (Telephone)	
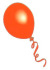
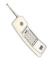
Lámpara (Lamp)		Zapato (Shoe)	
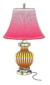
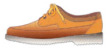

**Table A3:** List of operations used in the arithmetic task.

Additions					Subtractions				
16 + 6	38 + 8	52 + 2	61 + 5	75 + 6	11 − 3	36 − 4	45 − 2	58 − 1	75 − 6
17 + 4	39 + 3	52 + 4	62 + 7	75 + 9	12 − 8	36 − 5	45 − 4	61 − 5	76 − 4
18 + 1	41 + 2	53 + 3	64 + 3	77 + 5	17 − 4	37 − 8	48 − 2	62 − 7	83 − 8
18 + 8	42 + 3	54 + 2	65 + 1	83 + 8	18 − 1	38 − 1	48 − 7	64 − 3	84 − 5
19 + 2	42 + 4	54 + 3	65 + 2	84 + 5	19 − 2	39 − 1	51 − 2	65 − 1	94 − 3
26 + 2	43 + 8	55 + 2	66 + 3	94 + 3	26 − 2	39 − 3	52 − 4	65 − 2	96 − 1
33 + 6	45 + 2	55 + 6	67 + 1	97 + 2	33 − 6	41 − 2	54 − 2	66 − 3	96 − 4
34 + 3	45 + 4	56 + 9	67 + 7	97 + 5	33 − 7	41 − 7	54 − 3	69 − 4	97 − 2
35 + 4	48 + 5	57 + 6	69 + 4	97 + 8	34 − 3	42 − 3	54 − 7	69 − 8	97 − 5
36 + 1	48 + 7	58 + 1	69 + 8	98 + 3	35 − 4	42 − 4	55 − 2	71 − 9	97 − 8
36 + 5	49 + 3	59 + 5	73 + 6	99 + 7	35 − 7	42 − 8	56 − 9	73 − 6	98 − 3
37 + 8	51 + 2	61 + 1	73 + 9	99 + 8	36 − 1	43 − 8	57 − 6	73 − 9	99 − 8

## Appendix B. Analysis Complement

**Table A4:** Results from the ANOVA for reaction time in Trial 2.

Effect	*SS*	*df*	*MS*	*F*	*p*	*η* ^2^
SENTENCE	21,453	1	21,453	3.46	0.068	0.06
Residuals	303,147	49	6187			
OPERATION	235,407	1	235,407	14.47	<0.001	0.22
Residuals	797,140	49	16,268			
DISTANCE	112,478	1	112,478	13.5	<0.001	0.21
Residuals	408,258	49	8332			
SENTENCE × OPERATION	1231	1	1231	0.20	0.651	0.00
Residuals	290,933	49	5937			
SENTENCE × DISTANCE	2167	1	2167	0.38	0.536	0.00
Residuals	274,029	49	5592			
OPERATION × DISTANCE	114,800	1	114,800	20.10	<0.001	0.29
Residuals	279,922	49	5713			
SENTENCE × OPERATION × DISTANCE	112,633	1	112,633	12.28	<0.001	0.20
Residuals	449,402	49	9171			

SS = Sum of Squares, df = Degrees of Freedom, MS = Mean Squares, F = value of F, p = Probability, η^2^ = Eta squared.

**Table A5:** Results from the ANOVA for accuracy in Trial 2.

Effect	*SS*	*df*	*MS*	*F*	*p*	*η* ^2^
SENTENCE	0.38	1	0.38	0.06	0.803	0.001
Residuals	293.63	49	5.99			
OPERATION	783.6	1	783.6	30.64	<0.001	0.38
Residuals	1253.1	49	25.6			
DISTANCE	927.2	1	927.2	44.05	<0.001	0.47
Residuals	1031.4	49	21			
SENTENCE × OPERATION	34.8	1	34.8	5.17	0.027	0.09
Residuals	329.4	49	6.72			
SENTENCE × DISTANCE	0.01	1	0.01	<1	0.961	0.001
Residuals	387.5	49	7.91			
OPERATION × DISTANCE	184.9	1	184.9	12.65	<0.001	0.20
Residuals	716.1	49	14.61			
SENTENCE × OPERATION × DISTANCE	35.27	1	35.27	5.74	0.020	0.10
Residuals	300.83	49	6.14			

SS = Sum of Squares, df = Degrees of Freedom, MS = Mean Squares, F = value of F, p = Probability, η^2^ = Eta squared.

**Table A6:** Mean reaction times (in milliseconds) and accuracy (in %) across conditions.

Numerical Distance	Close				Far			
**Type of operation**	Additions		Subtractions		Additions		Subtractions	
**Type of sentence**	Inc.	Dec.	Inc.	Dec.	Inc.	Dec.	Inc.	Dec.
**Reaction time**	825	881	944	926	863	843	848	888
	(56.2)	(56.2)	(56.2)	(56.2)	(56.2)	(56.2)	(56.2)	(56.2)
**Accuracy**	96.3	95.1	90.9	92.2	97.4	97.4	95.9	96.0
	(0.77)	(0.77)	(0.77)	(0.77)	(0.77)	(0.77)	(0.77)	(0.77)

Standard errors are reported in brackets. Inc.: Increase, Dec.: Decrease.
